# Development and Characterization of Rice Husk and Recycled Polypropylene Composite Filaments for 3D Printing

**DOI:** 10.3390/polym13071067

**Published:** 2021-03-28

**Authors:** Maria A. Morales, Cindy L. Atencio Martinez, Alejandro Maranon, Camilo Hernandez, Veronique Michaud, Alicia Porras

**Affiliations:** 1 Grupo de Diseño de Productos y Procesos (GDPP), Department of Chemical and Food Engineering, Universidad de los Andes, CR 1 18a 12, Bogotá 111711, Colombia; ma.morales12@uniandes.edu.co (M.A.M.); cl.atencio@uniandes.edu.co (C.L.A.M.); 2 Structural Integrity Research Group, Department of Mechanical Engineering, Universidad de los Andes, CR 1 18a 12, Bogotá 111711, Colombia; emaranon@uniandes.edu.co; 3 Sustainable Design in Mechanical Engineering Research Group (DSIM), Department of Mechanical Engineering, Escuela Colombiana de Ingenieria Julio Graravito, Autopista Norte AK 45 205 59, Bogotá 111166, Colombia; camilo.hernandez@escuelaing.edu.co; 4 Laboratory for Processing for Advanced Composited (LPAC), Institute of Materials, Ecole Polytechnique Fédérale de Lausanne (EPFL), EPFL-STI-IMX-LPAC, Station 12, CH-1015 Lausanne, Switzerland; veronique.michaud@epfl.ch

**Keywords:** composites, rice husk, recycled polypropylene, 3D printing, fused filament fabrication

## Abstract

Nowadays the use of natural fiber composites has gained significant interest due to their low density, high availability, and low cost. The present study explores the development of sustainable 3D printing filaments based on rice husk (RH), an agricultural residue, and recycled polypropylene (rPP) and the influence of fiber weight ratio on physical, thermal, mechanical, and morphological properties of 3D printing parts. Thermogravimetric analysis revealed that the composite’s degradation process started earlier than for the neat rPP due to the lignocellulosic fiber components. Mechanical tests showed that tensile strength increased when using a raster angle of 0° than specimens printed at 90°, due to the weaker inter-layer bonding compared to in-layer. Furthermore, inter layer bonding tensile strength was similar for all tested materials. Scanning electron microscope (SEM) images revealed the limited interaction between the untreated fiber and matrix, which led to reduced tensile properties. However, during the printing process, composites presented lower warping than printed neat rPP. Thus, 3D printable ecofriendly natural fiber composite filaments with low density and low cost can be developed and used for 3D printing applications, contributing to reduce the impact of plastic and agricultural waste.

## 1. Introduction

The fourth industrial revolution allows alternative industrial models where materials are repurposed for further uses taking advantage of digital technologies [1]. Some of these materials are plastic waste sent to be recycled [2] and agricultural residues [3]. One of those technologies is 3D printing [4].

In particular, plastic waste has increased dramatically since polymers’ industrial production started in the 20th century: 79% of plastics produced to date has been accumulated in dumps or landfilled [5], remaining in the environment for extended periods [6,7,8,9], even contaminating the food chain [10,11,12,13]. Therefore, developing practical and sustainable alternatives to reduce such waste has attracted significant interest by producing parts from recycled resources [14,15,16]. Moreover, agricultural residues are becoming an environmental problem as only a minimal fraction of the 155 billion tons/year of organic matter production from the photosynthetic process can be consumed directly by humans or animals [17]. Residues become waste as they do not have commercial use [17,18,19], affecting human health and damaging the environment [20,21,22]. Therefore, the safe disposition or recycling of agricultural waste has been considered paramount in the green economy [23,24,25,26].

On the other hand, 3D printing is a rapidly evolving digital technology [27,28], enabling the automatic fabrication of complex shapes with minimum material waste [29,30] for many applications such as automotive, aerospace, construction, and medicine [31,32,33,34]. Among 3D printing technologies, the Fused Filament Fabrication (FFF) is the least expensive, producing parts using multiple different materials [30,35,36]. In the FFF process, a polymeric filament is continuously fed into a heated nozzle that melts the polymer, layering it in thin slabs onto a print bed, forming a 3D part [37,38,39,40]. Commonly, amorphous or low crystallinity thermoplastics, such as ABS, nylon, or PLA, are used as feedstock materials for FFF [41,42,43], as they exhibit a low degree of warping, which contributes to dimensional accuracy [44]. 

Recently, agricultural residues have been used as a loading material for 3D printing [45,46,47,48,49,50,51,52,53] as they reduce warping [54,55,56,57], enhance printing directionality [58,59,60], and display high specific mechanical properties [61,62,63]. However, in FFF there are some standing issues such as residues’ agglomeration, viscosity variations, high porosity, low thermal stability [64], and non-uniform physical and mechanical properties [61]. One particular example of these residues is rice husk (RH), which most accumulates in Colombia, reaching about 63 tons/day [20]. Studies using RH as load material have found that the mechanical properties of samples are affected by the printing direction [59], that adding methylene diphenyl diisocyanate improves the mechanical performance reducing the presence of voids [65], and that acrylic acid grafting on PLA enhances tensile and physical properties of PLA/RH printed specimens [66].

Moreover, there is a growing interest in using amorphous and low-crystalline recycled plastics, like PLA [67] and PET [68], as feedstock materials for printing biocomposites. However, the use of semi-crystalline recycled plastics, such as polypropylene, has been limited [69,70,71] due to the printed parts’ tendency to warp and shrink [72,73]. In Colombia, polypropylene is produced at about 500,000 tons/year [74], and it is one of the major contributors to marine and urban pollution [75].

Although natural fibers-filled polymers are gaining importance in the 3D printing market and remarkably growing interest from the scientific community, several new agro-waste natural fiber sources and polymer combinations remain to explore their potential structural and economic performance in FFF applications. In particular, rice husk reinforced recycled polypropylene composite is a novel feedstock for 3D printing applications. Therefore, in this work, rice husk and recycled polypropylene are employed to develop and characterize a sustainable composite filament for use in the Fused Filament Fabrication process. The manufacturing process of the composite filament is fist described. Physical, mechanical, thermal, and morphological properties of 3D printed specimens using this novel composite filament are characterized. Density, water absorption, thermogravimetric analysis, tensile test, and electronic microscopy were used to explore printed structures properties with different rice husk content (fiber weight ratio). Additionally, different printing raster angles were employed to explore the variation of mechanical properties with printing direction. All properties were compared against the benchmark neat recycled PP.

## 2. Materials and Methods

### 2.1. Materials

Promaplast S.A.S. supplied recycled polypropylene (rPP) pellets from post industrial waste.

Rice husk was obtained from Ambala grinder from the Tolima Department, in Colombia. 

### 2.2. Extrusion of Composite Filaments

Pulverisette 19 mill was used to grind the RH to reduce particles’ size, to reach a particle size between 250 and 425 μm. Before mixing, pulverized RH and rPP pellets were dried at 105 °C for three hours. Compounds were prepared by weighing RH fiber and rPP to obtain 0, 5 and 10 wt.% fiber content, and hand mixing before feeding to the extruder. 

**Figure 1:**
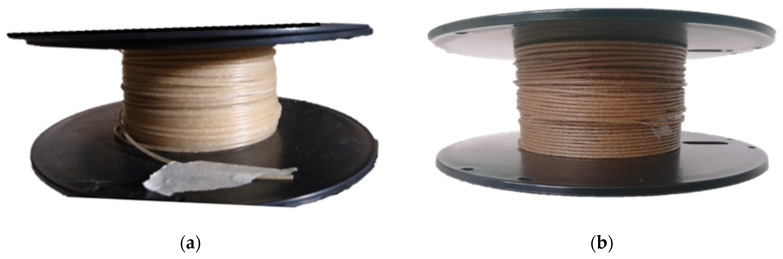
rPP/RH 3D printing filaments with (**a**) 5 wt.% and (**b**) 10 wt.% of RH.

A Brabender DSE 20 twin extruder with six temperature-controlled zones was used for extrusion. The feed port was fixed at 180 °C and the adjacent zone to 185 °C. The following two zones were fixed at 190 °C. The last zone was set at 195 °C and the die at 195 °C. Screw speed was maintained at 9 rpm. The extruded filament was collected on water at a temperature of 25 °C. The resulting material was granulated in a pelletizer and re-extruded to improve homogenization of the mixture. Extruder parameters for the second extrusion were set at the same values as the first. A 2 mm diameter nozzle was used to generate filaments with a target diameter of 1.75 ± 0.05 mm (Figure 1).

### 2.3. Density

Density of rPP and rPP/RH composites was measured following the ASTM D792, test method B, using ethanol (ρ = 0.789 g/cm^3^) at 19.9 °C as immersion liquid. An electronic balance was used to weigh the specimens. Three specimens were tested per material.

### 2.4. Water Absorption and Diameter Swelling Test

Three specimens per material (length = 50 ± 0.1 mm) were prepared according to the ASTM D570 standard to test the percentage increase in weight. Preconditioning of the specimens was carried out, specimens were dried at 50 °C for 24h, cooled in a desiccator, and immediately weighted (W_0_). Later specimens were immersed in distilled water for 2h at room temperature, all surface water wiped off with a dry cloth, and weighed (W_i_). The weight percentage increase during the immersion, was calculated using Equation (1).

Increase in weight (weight %) = (W_i_ − W_0_)/W_0_ × 100 (1)

The swelling diameter of the composite was determined by measuring the diameter of each specimen before (D_0_) and after (D_i_) water immersion test, according to Equation (2).

Diameter swelling (%) = (D_i_ − D_0_)/D_0_ × 100 (2)

### 2.5. Thermal Analysis

A thermogravimetric analyzer (SDT Q600) was used to characterize the composites’ thermal stability and the neat polymer, subjected to the same extrusion process as the composites. Measurements were performed in nitrogen atmosphere with sample weight of 2 mg according to the ASTM E1131 standard. Samples were heated from ambient temperature to 700 °C at a rate of 10 °C/min. Further thermal analysis was carried out according to the ASTM D3418 standard using a DSC Q2000. First, sample temperature was increased from the ambient temperature to 220 °C, at a heating rate of 10 °C/min and held in an isothermal state for 5 min, to eliminate thermal history, residual moisture, and voids. Then, the sample was cooled down to room temperature at 10 °C/min, and reheated to 220 °C at 10 °C/min. The samples’ degree of crystallinity was calculated by Equation (3) [69,76,77], considering the polymer fraction in the material [78,79,80].

% crystallinity = (ΔH_f_^obs^/ΔH_f_^0^) × 100/(100 − w^f^) (3)

where ΔH_f_^obs^ is the observed enthalpy of fusion, ΔH_f_^0^ the heat of fusion of the completely crystalline materials at the equilibrium melting temperature T_m_ (207 J/g [81,82,83]) and w^f^ is the fiber weight ratio in composites.

### 2.6. Specimen Manufacturing

**Figure 2:**
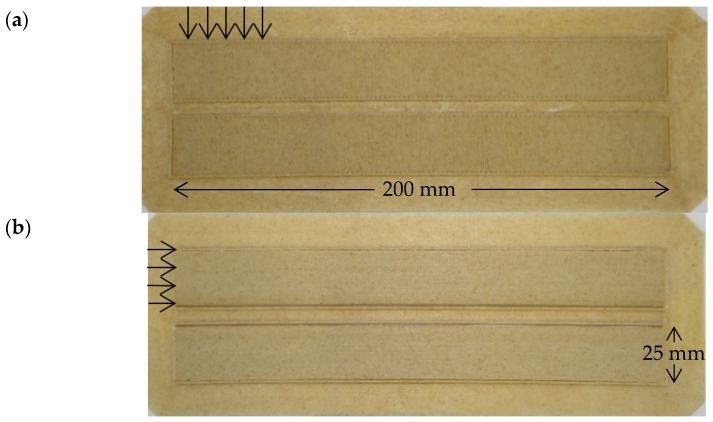
3D printing tensile specimens at (**a**) 90° and (**b**) 0° with brim platform.

According to the ASTM D3039/3039-08 standard (200 mm × 25 mm × 2.5 mm) on a 3D FF STD Doppia machine, the filament was printed into tensile specimens. STL files were imported into Simplify3D for editing. The filament was printed on a Magigoo 3D Printing Adhesive for PPGF using a brim platform to avoid warping effects for all samples. Bed temperature for the first layer was set to 90 °C, and 70 °C for the rest of the layers. Nozzle temperature was set to 240 °C. The layer height was set to 0.25 mm, with a nozzle diameter of 0.8 mm, 100% infill, and a printing speed of 60 mm/s. Two types of specimens were printed at different raster angles, 0° and 90°, to determine the tensile properties as a function of the raster angle. 3D printed specimens are shown in Figure 2.

### 2.7. Mechanical Measurements

Uniaxial tensile tests were conducted on an Instron 3367 universal testing machine, equipped with a 30 kN load cell according to the ASTM D3039/3039 standard. Five tensile specimens were tested per composition and print conditions until failure. Tensile tests were performed at a gauge length of 50 mm and a crosshead speed of 10 mm/min, and 1.2 mm/min for specimens printed at 90° and 0° respectively. Strain was measured for all specimens using an extensometer fixed to the samples. 

### 2.8. Statistical Analysis

Physical and mechanical properties of materials are influenced by different parameters, including fiber weight ratio. Analysis of variance is the central technique in the experimental data analysis. It consists of dividing the total variation observed in each of the sources that contribute to it. ANOVA assesses the importance of one or more factors by comparing the response variable means at the different factor levels [84].

In this study, a one-way analysis of variance was carried out, considering the fiber content as the factor, with three levels (0 wt.%, 5 wt.% and 10 wt.%). Three specimens were evaluated for density, and five specimens were tensile tested for each configuration. Density, water absorption, tensile strength and Young’s modulus were used as analysis criteria (response variable). To this purpose, a *p*-value of *p* < 0.05 was considered statistically significant. Minitab 18 Statistical Software was used to analyze data. An analysis of variance was carried out for each 3D printing raster angle. 

### 2.9. Scanning Electron Microscopy Measurements

Fractures of failed tensile composites specimens were observed using a JEOL SEM JSM-6490LV. Specimens were prepared by gold-sputtering for 1 min at 20 mA to obtain good conductivity. 

## 3. Results and Discussion

### 3.1. Physical Properties

Table 1 reports density values for the neat rPP and the composites. As expected, the density decreased with increasing fiber content and showed small variations. This decrease is due to the lower density of the filler fiber (between 0.88 and 0.12 g/cm^3^) as compared to rPP (0.89 g/cm^3^) [59,85]. The rPP with 10 wt.% of RH showed a density decrease of 2.0% as compared to rPP. This result is consistent with other results reported for wood apple shell reinforced epoxy composites [86], foams reinforced with cassava starch [87], and foams reinforce with cassava/sugar palm [88]. This density reduction is greater (up to 3.8%) if the results are compared against neat PP (which has a slightly higher density than rPP, at 0.91 ± 0.01 g/cm^3^) [89]. According to the statistical analysis with a significance of 5%, the fiber weight ratio has a significant impact on the density of composite materials. Further analysis was made with a Tukey test, obtaining a significant difference between rPP and rPP/RH (10 wt.%) composite densities. 

Low-density composites are widely used in different industries. For example, the lower overall weight of composites based on natural fibers is advantageous in the automotive industry since light parts increase fuel efficiency and increase the sustainability of the manufacturing process [90,91,92]. 

**Figure 3:**
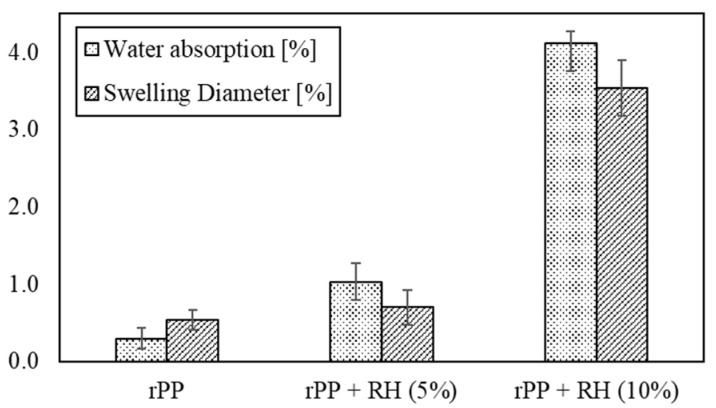
Water absorption and swelling diameter of rPP and rPP/RH composites.

Water absorption and diameter swelling of the filaments were measured to compare the hydrophobic behavior of rPP and rPP/RH composites. As shown in Figure 3., rPP/RH composites have a higher water absorption and a higher swelling diameter due to natural fibers’ hydrophilic behaviors [29,66,93,94,95]. These parameters increase with fiber weight ratio. Razavi et al. investigated the water absorption behavior of chopper rice husk-filled polypropylene composites manufactured through injection molding, his study showed that a higher weight fiber increases water absorption, reaching about 300% with 10 wt.% of RH, compared to neat PP [96]. Obtained results for composites were similar to reported values obtained for PLA and PHA and natural fibers composites [45,97]. The importance of drying the fibers well before the composites processing, lies in the fact that natural fibers’ moisture and swelling behavior induce debonding between fibers and matrix [98,99,100,101,102,103,104]. Moreover, a reduction in mechanical properties and degradation temperature has been observed due to high level moisture absorption by natural fibers [98,105,106]. Improvement in water absorption and swelling diameter could be reached by surface treatment [98,105,107].

### 3.2. Thermal Analysis

**Figure 4:**
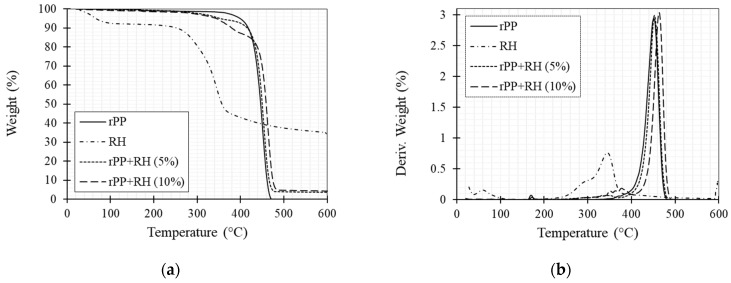
(**a**) TGA and (**b**) DTGA curves of RH fiber, rPP and rPP/RH composite filaments obtained using TGA.

Thermal stability of RH fiber, neat rPP, rPP/RH (5 wt.%) and rPP/RH (10 wt.%) was studied by thermo-gravimetric analysis. Figure 4 shows the percentage weight change and the derivative of weight as a function of each material’s temperature. For the RH fibers, three stationary regimes are observed. A first phase ranging from 50 to 100 °C is linked to the moisture content of the fiber, representing 10% of the weight; the second phase, with a significant decay at 280 °C is related to the degradation of hemicelluloses and partial decomposition of lignin, and the third point at 340 °C is associated with α-cellulose and remaining lignin degradation [108]. The observed thermal behavior of RH was similar to that of other lignocellulosic fibers [19,29,109]. For the rPP curves, a small weight change is observed between 165 and 175 °C, attributed to impurities’ presence due to the recycled nature of the polymer. Moreover, between 400 to 490 °C a main step is associated with the main degradation of the polypropylene. rPP/RH (5 and 10 wt.%) curves present combined loss behavior. The first step ranging from 50 to 120 °C is attributed to the evaporation of moisture. The next two steps represent the fiber components degradation, and the last represents the matrix degradation at 460 °C. Finally, for RH and rPP/RH (5 and 10 wt.%) composites, the residual mass percentage at 600 °C was around 34.45%, 3.52%, and 3.88%, respectively.

In summary, these results show that fiber thermal degradation may occur during the printing process with an extruder temperature above 280°C. However, with adequate printing temperature, rPP/RH composites stability is a priori suitable for 3D printing applications.

**Figure 5:**
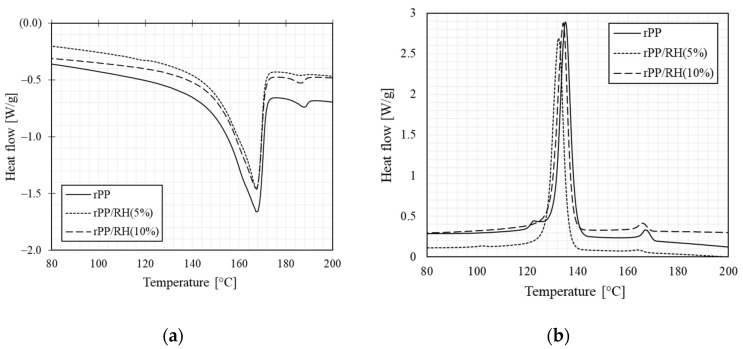
(**a**) DSC melting and (**b**) crystallization thermograms of rPP and rPP/RH composites.

Thermal phase transitions of the samples were measured with DSC. Figure 5 shows the crystallization and melting transition curves of rPP and rPP/RH (5 and 10 wt.%). In Figure 5a, endotherms show that the rPP has a well-defined melt transition centered at 167.27 °C. The phase transformation occurs between 130 and 175 °C. The melting behavior of the composites is very similar to that of rPP. The melting endotherm display a small peak at around 185°C, which can be attributed to impurities due to its recycled nature.

Exotherms shown in Figure 5b indicates that the crystallization temperature of rPP is 135.07 °C. Phase transformation occurs between 125 and 145 °C. The composite materials’ corresponding crystallization temperatures decrease to 132 and 134 °C for rPP/RH (5%) and rPP/RH (10%), respectively. These temperatures suggest a lower crystallization rate [110], than the neat rPP (135 °C). This feature is crucial as it may imply a decrease of the warping effect during the composites’ printing process compared to the neat rPP.

RH’s presence in the rPP matrix is known to interact with the crystallization process of the rPP and decrease its crystallization temperature. As shown in Table 2, this may be caused by RH’s inhibition effect on the rPP crystal formation [111,112]. 

On the other hand, when increasing the fiber fraction from 5 to 10 wt.%, a slight increase of the crystallinity, from 37.62 to 43.23%, is observed. It can point to the possibility that RH fiber, if in sufficient quantity, acts as nucleating sites, causing the acceleration of the crystallization of the composite [109,113].

### 3.3. Mechanical Properties

**Figure 6:**
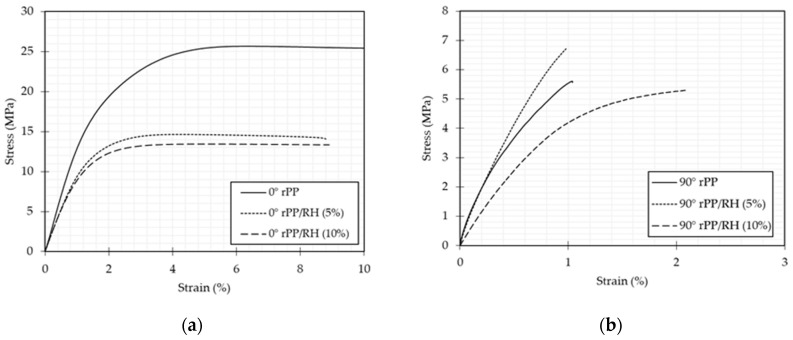
Stress-strain curve for rPP and rPP/RH (5 and 10 wt.%) composites (**a**) printed at 0° and (**b**) printed at 90°.

During the 3D printing of tensile specimens, as speculated with thermal results, composites exhibited less warping effect than neat rPP. Figure 6 shows the representative stress–strain curves for 3D printed tensile specimens at 0° and 90° of rPP and rPP/RH composites with 5% and 10 wt.% RH. These curves show an initial linear elastic region followed by plastic deformation up to failure. Comparing the raster angle evaluated, curves for rPP/RH composites at different fiber weight ratios show similar behaviors, which is an advantage in fiber implementation and potential variability. For specific applications of 3D printing filaments, the use of lighter components is preferable. In this case, adding 10 wt.% of fiber does not significatively affect the strain to failure, denoting that 10 wt.% of fiber can be used to develop of 3D printing filaments and prototypes.

However, for 0° specimens, neat rPP is stiffer and has a significantly higher toughness than the composites, as evaluated from the area under the curves, showing that it is more susceptible to dissipate energy. Instead, for 90° angle rPP/RH (5%) are stiffer than neat rPP and rPP/RH (10%) and has better ultimate strength. Moreover, for 90° rPP/RH (10%) an increase in toughness is observed. Even though, the mechanical properties of rPP/RH composites are in general lower than those of the neat rPP, results demonstrate that rice husk can be considered as a potential filler since using a raster angle of 0°, there is no significant difference in mechanical properties between 5 and 10 wt.% of fiber. Besides, using 10 wt.% of rice husk and a raster angle of 90°, the material is more ductile and has a higher energy absorption capacity.

Table 3 summarizes the tensile properties of rPP and rPP/RH composites printed at different raster angles. The printed specimens’ orthotropic nature is attributed to the difference between bonding mechanisms at 0° and 90° of raster angle, as the inter-layer connection is weaker than in-layer [114]. Guen et al. [59] also observed the orthotropy-induced difference in properties between the lengthwise (0°) and widthwise (90°) printing raster angle. When a tensile test is carried out in specimens printed at 90°, the main mechanism of failure is the detachment of layers. In other words, the adhesion between layers is evaluated in these tests. Whereas with the tensile test of specimens printed at 0°, the intrinsic material properties are evaluated. 

Before data analysis, a statistical analysis was performed. For specimens printed at 0° and 90°, the fiber weight ratio is significant in tensile strength and tensile elongation. Moreover, by a Tukey comparison, it was demonstrated that tensile strength is not significantly different between both composites. 

A potential explanation for the loss in tensile strength of the composites as compared to the neat rPP in the 0° direction is the absence of chemical bonding between rPP and RH and the low dispersion of RH on the rPP matrix due to the large differences in the surface energies of the rPP and RH filler [65,108]. Osman et al. tested natural fiber 3D printed composites based on ABS and rice straw at 0°. They also reported a loss in tensile strength with fiber addition, by 31.61% from the neat ABS to ABS and 10 wt.% of rice straw [115]. In general, comparing unfilled and natural fiber composite printed parts, it is often concluded fiber’s addition has a negative influence on strength [55,111,116,117,118,119]. Even for composites produced by compression molding, a similar effect is reported by Rice et al. for polypropylene composites made with 20% of rice husk. The tensile strength decreases by almost 20% compared to neat polypropylene [19]. Nevertheless, compared with the neat rPP, the composite material has the advantages of a reduced cost and easy processability in reducing of the warping during the 3D printing process. Moreover, it is important to highlight its lower density for light material application, as in concept parts, lightweight custom jigs and fixtures or thermoformed parts, and the sustainable nature due to the use of recycled polymers and natural fibers for its development.

**Figure 7:**
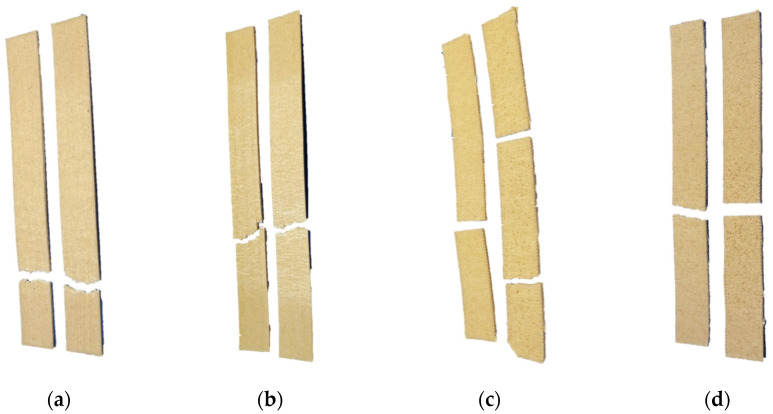
Tensile test fractured specimens. (**a**) rPP/RH 5 wt.% at 0°: Angled gage bottom (AGB) failure mode; (**b**) rPP/RH 10 wt.% at 0°: Angled gage middle (AGM) failure mode; (**c**) rPP/RH 5 wt.% at 90°: Lateral gage middle (LGM) failure mode; and (**d**) rPP/RH 10 wt.% at 90°: Lateral gage middle (LGM) failure mode.

Figure 7 shows tensile test failure mode of a couple of specimens of each configuration. The fracture mode depends on the raster angle used. For the 0° angle, presented in Figure 7a,b, fracture occurred perpendicular to the layer’s deposition direction with an irregular fracture. According to the failure codes proposed by the ASTM D3039 standard, composite specimens with 5 wt.% of RH tended to fail with an angle at the bottom grip (AGB). Meanwhile those with 10 wt.% of fiber failed angled in the middle of the specimen (AGM). Those specimens show some ductile mode of failure. An amount of deformation is observed in each 3D printed layer. For the 90° raster angle specimens, shown in Figure 7c,d, the failure occurs through the bonded layers, adjacent to the layers’ deposition direction, in a more brittle manner and in the gauge region. The brittle fracture mode of specimens printed at 90° is due to the poorly bonding between in-dividual printed layers [120]. According to the standard, the failure occurred lateral at the gage in the middle (LGM) for both specimens, with 5 and 10wt.% of RH. During the test, some specimens showed a premature fracture due to stress concentration areas caused by imperfections during the printing process. These imperfections were produced due to fluctuating diameter; this trouble was already reported [121,122].

### 3.4. Tensile Test Specimens Fracture Morphology

**Figure 8:**
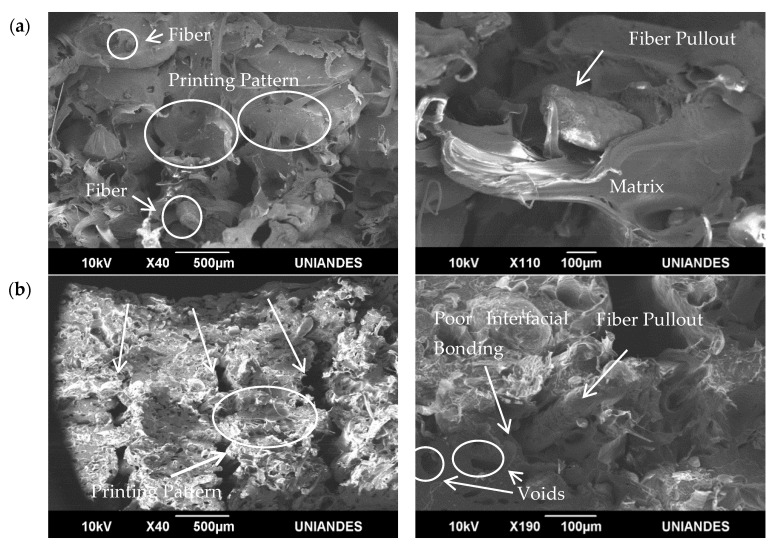

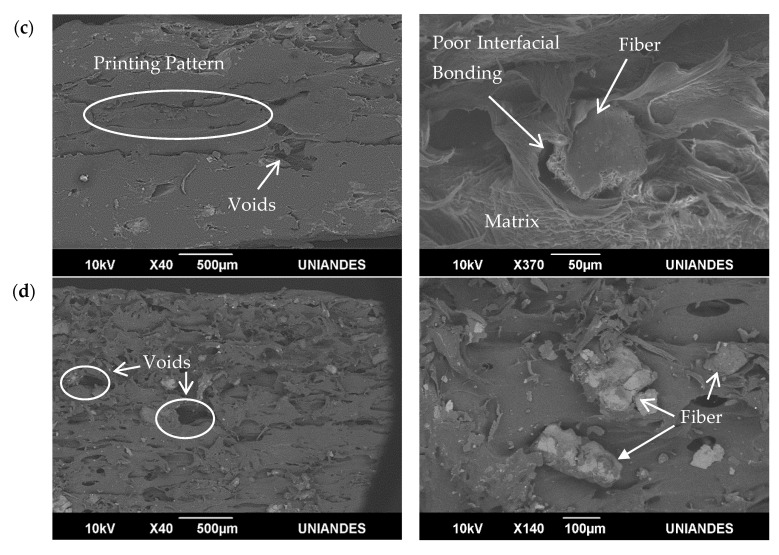
SEM images of tensile fractured specimens. (**a**) rPP/RH 5 wt.%. at 0°; (**b**) rPP/RH 10 wt.%. at 0°; (**c**) rPP/RH 5 wt.%. at 90° and (**d**) rPP/RH 10 wt.% at 90°. All the images were acquired at different magnifications in BEC mode.

SEM micrographs of the cross-sectional fracture surfaces of the printed rPP/RH composite specimens with different raster angles and compositions are shown at different magnification levels in Figure 8. Figure 8a,b represent printed specimens at 0°, with 5 and 10 wt.% of RH, respectively. Lengthwise printing pattern can be identified by the filament’s cylindrical geometry and the gaps between layers due the poor adhesion. At higher magnification, fiber pull-out and matrix breakage are observed as the main failure mechanism. Non-uniform dispersion of RH in the rPP matrix is observed due to RH particles’ irregular morphology and a weak adhesion between the RH and the rPP matrix. This poor interfacial bonding between the RH and rPP and RH agglomerates’ presence can be attributed to the low polarity of rPP and the high energy of RH [123]. Filler agglomeration decreased the composite homogeneity and resulted in the void formation, which acted as stress concentrators. These stress concentrators led to the composites’ early rupture due to the nonuniform stress [19,124]; this could explain why the specimens’ tensile strength drops significantly when RH content is added higher. It is important to highlight that for plant-based composites the limited interaction between the hydrophilic fibers and hydrophobic matrices is generally observed [125]. Physical and mechanical fiber treatment can improve the interfacial bonding between the fiber and the matrix [71,125,126,127]. The most common chemical treatment is the alkali treatment [128]. During this treatment, the lignin, wax and oil covering natural fibers are removed, increasing the fiber surface’s roughness [129]. Reactive coupling agents are also added to improve adhesion between the fiber and the polymer matrix [130].

Figure 8c,d show printed specimens at 90° with 5 and 10 wt.% of RH, respectively. Figure 8c shows a mostly layered surface, with a smooth finish in each layer. In these micrographs the specimen failure was attributed to interlayer bonding. The surface is clean, and it does not show any pull-out fibers or holes due to this failure mechanism. However, voids between each layer are observed due to the presence of air between layers during 3D printing process. Moreover, in 3D printing the deposition of molten filaments adjacent to solid layers results in poor interfaces [131]. By contrast, Figure 8d shows fiber and matrix breakage as the main failure mechanism, as the broken fibers and fillers are more easily identified.

## 4. Conclusions

In this research, rice husk and recycled polypropylene composite filaments for 3D printing were successfully manufactured and characterized using different rice husk weight ratios and raster angles. The addition of fiber to the neat rPP decreases the warping produced during the printing process. The composite density decreases as the fiber weight ratio increases due to the low density of the fiber. Water absorption and swelling diameter increase in the rPP/RH composites because natural fibers have a hydrophilic behavior. Thermal analysis showed that for rPP/RH composites the degradation process started earlier than for the neat rPP due to the fiber’s lignocellulosic components, though it does not affect its capability to be printed. On the other hand, the crystallinity increases in the composite material, between 5 to 10 wt.%, because fiber may act as nucleating sites.

Regarding the mechanical properties, the tensile strength decreases using a raster printing angle of 90° compared to the 0° printing raster angle. This decrease is due to the weak interlayer bonding. Even though mechanical properties decrease significantly with the introduction of RH in the polymer, they are still attractive for different applications where these mechanical properties are sufficient, as concept parts, lightweight custom jigs and fixtures, and thermoformed parts. Differences between 5 and 10 wt.% are not significant, making rice husk fiber a good filler to be used at 10 wt.%, to take advantage of this agro-industrial waste. Moreover, more fiber content makes the material lighter and more cost-effective than commercial filaments.

SEM micrographs of composite materials confirm the limited interaction between the untreated fiber and rPP matrix related to the reduced strength.

This novel natural rice husk and recycled polypropylene composite material designed as a feedstock filament for 3D printing was evaluated physically, mechanically, and thermally. These properties are useful information to other researchers or end-users to manufacture eco-friendly filaments or printed parts. Based on these results, it can be concluded that 3D printable natural fiber composites based on recycled polymers exhibit low density and low cost, and thus have the potential to be manufactured and find applications in appropriate fields. Natural fibers and recycled polymers make the material sustainable, helping us coming closer to a circular economy.

## Figures and Tables

**Table 1:** Density of neat rPP and rPP/RH composites.

Sample	Density (g/cm^3^)
rPP	0.893 ± 0.001
rPP/RH (5%)	0.883 ± 0.004
rPP/RH (10%)	0.876 ± 0.004

**Table 2:** Temperatures and enthalpies of fusion and crystallization of rPP and rPP/RH composites.

Sample	Crystallization		Melting		%Crystallinity
	T_c_ (°C)	ΔH_c_ (J/g)	T_m_ (°C)	ΔH_m_ (J/g)	Based on ΔH_PP_^0^
rPP	135.07	84.28	167.27	94.83	45.81
rPP/RH (5 wt.%)	132.39	81.01	167.39	73.98	37.62
rPP/RH (10 wt.%)	134.26	84.63	167.24	80.54	43.23

**Table 3:** Mechanical properties of rPP and rPP/RH composites at different raster angles.

Raster Angle	Specimen	Tensile Strength (MPa)	Tensile Elongation (%)	Young Modulus (GPa)
0°	rPP	26.02 ± 0.47	6.16 ± 0.19	1.34 ± 0.05
	rPP/RH (5 wt.%)	13.62 ± 2.71	4.10 ± 0.20	1.06 ± 0.13
	rPP/RH (10 wt.%)	13.78 ± 0.59	5.06 ± 0.16	1.04 ± 0.04
90°	rPP	4.33 ± 1.73	1.01 ± 0.35	0.74 ± 0.37
	rPP/RH (5 wt.%)	7.92 ± 0.67	2.04 ± 0.43	1.01 ± 0.12
	rPP/RH (10 wt.%)	5.66 ± 0.82	3.07 ± 0.45	0.66 ± 0.13

## Data Availability

The data presented in this study are available upon request from the corresponding author.
